# High relative amount of nodular calcification in femoral plaques is associated with milder lower extremity arterial disease

**DOI:** 10.1186/s12872-022-02945-7

**Published:** 2022-12-23

**Authors:** Mae Azeez, Mirjami Laivuori, Johanna Tolva, Nina Linder, Johan Lundin, Anders Albäck, Maarit Venermo, Mikko I. Mäyränpää, Marja-Liisa Lokki, A. Inkeri Lokki, Juha Sinisalo

**Affiliations:** 1 Transplantation Laboratory, Department of Pathology, University of Helsinki, Haartmaninkatu 3, FIN-00014 Helsinki, Finland; 2 Department of Vascular Surgery, Helsinki University Hospital and University of Helsinki, Haartmaninkatu 4, PL 340, FIN-00029 HUS, Helsinki, Finland; 3 Institute for Molecular Medicine Finland, HILIFE, University of Helsinki, FIN-00014 Helsinki, Finland; 4 Present Address: Department of Public Health Sciences, Global Health/IHCAR, Karolinska Institutet, SE-171 77 Stockholm, Sweden; 5 Department of Pathology, University of Helsinki and Helsinki University Hospital, Haartmaninkatu 3, FIN-00014 Helsinki, Finland; 6 Translational Immunology Research Program, Research Programs Unit, University of Helsinki, Haartmaninkatu 8, FIN-00014 Helsinki, Finland; 7 Department of Cardiology, Heart and Lung Center, Helsinki University Hospital and University of Helsinki, Haartmaninkatu 4, PL 340, FIN-00029 HUS Helsinki, Finland

**Keywords:** Lower extremity arterial disease, Deep learning algorithm, Vascular calcification, Sheet calcification, Nodular calcification, Atherosclerosis

## Abstract

**Background:**

Clinical implications of different types of vascular calcification are poorly understood. The two most abundant forms of calcification, nodular and sheet calcification, have not been quantitatively analyzed in relation to the clinical presentation of lower extremity arterial disease (LEAD).

**Methods:**

The study analyzed 51 femoral artery plaques collected during femoral endarterectomy, characterized by the presence of > 90% stenosis. Comprehensive clinical data was obtained from patient records, including magnetic resonance angiography (MRA) images, toe pressure and ankle brachial index measurements and laboratory values. The plaques were longitudinally sectioned, stained with Hematoxylin and Eosin and digitized in a deep learning platform for quantification of the relative area of nodular and sheet calcification to the plaque section area. A deep learning artificial intelligence algorithm was designed and independently validated to reliably quantify nodular calcification and sheet calcification. Vessel measurements and quantity of each calcification category was compared to the risk factors and clinical presentation.

**Results:**

On average, > 90% stenosed vessels contained 22.4 ± 12.3% of nodular and 14.5 ± 11.8% of sheet calcification. Nodular calcification area proportion in lesions with > 90% stenosis is associated with reduced risk of critically low toe pressure (< 30 mmHg) (OR = 0.910, 95% CI = 0.835–0.992, *p* < 0.05), severely lowered ankle brachial index (< 0.4) (OR = 0.912, 95% CI = 0.84–0.986, *p* < 0.05), and semi-urgent operation (OR = 0.882, 95% CI = 0.797–0.976, *p* < 0.05). Sheet calcification did not show any significant association.

**Conclusions:**

Large amount of nodular calcification is associated with less severe LEAD. Patients with nodular calcification may have better flow reserves despite local obstruction.

**Supplementary Information:**

The online version contains supplementary material available at 10.1186/s12872-022-02945-7.

## Introduction

Atherosclerosis is the underlying cause of the current cardiovascular disease epidemic. It is estimated that > 200 million people suffer from peripheral arterial disease worldwide. The amount of calcification in lower limb artery plaques has been associated with higher cardiovascular morbidity and mortality [1, 2].

Vascular calcification is a continuum of osteoid metaplasia formation that is observed in various morphologies. The plaque histology is reflected on the clinical symptoms [3, 4]. Vascular calcification initiates as microcalcification that may over time progress into macrocalcification [5]. This is believed to have a stabilizing effect on the plaque [6]. Macrocalcification can occur in the form of a calcified plate, called sheet calcification or as osteoid metaplasia [4, 7]. The origin of nodular calcification is under debate. Calcified thrombi may give rise to nodular calcification. Fibrin deposits surrounding the nodular structures suggests thrombotic origin [8]. On the other hand, nodular calcification may occur at the sites of sheet calcification fractures [7, 9]. When a detached plaque fragment erupts through the protective cap of a plaque into the bloodstream, a thrombus forms around it, which is detected as fibrin surrounding the nodular calcification [10]. Histology is a valuable tool for understanding plaque composition and quantification of tissue structures. Previously, calcification in lower limb arteries and its clinical implications have been semi-quantified using imaging scoring [1, 2, 11]. To our knowledge, the quantity of nodular calcification and sheet calcification in femoral atherosclerosis and their impact on lower extremity arterial disease (LEAD) presentation has not been thus far analyzed.

Despite its dense fibrocalcific character, critical atherosclerotic obstruction of femoral artery less frequently has dramatic consequences than atherosclerotic obstruction of coronary and carotid arteries [12]. This is partly due to the larger size of the vessel but also the compensatory vascular remodeling observed in this artery, where plaque initially grows outwards [12, 13]. Previous studies have not compared the impact of different calcified morphologies on the clinical severity of LEAD. sss.

Therefore, in this study, we quantify nodular calcification and sheet calcification in femoral plaque sections. Our aim is to analyze and compare the area proportion of each calcification category to the LEAD severity.

We apply a deep learning algorithm to obtain a consistent measuring method across the studied histological plaque sections in order to obtain accurate quantification of calcification.

## Methods

### Cohort description

**Fig. 1:**
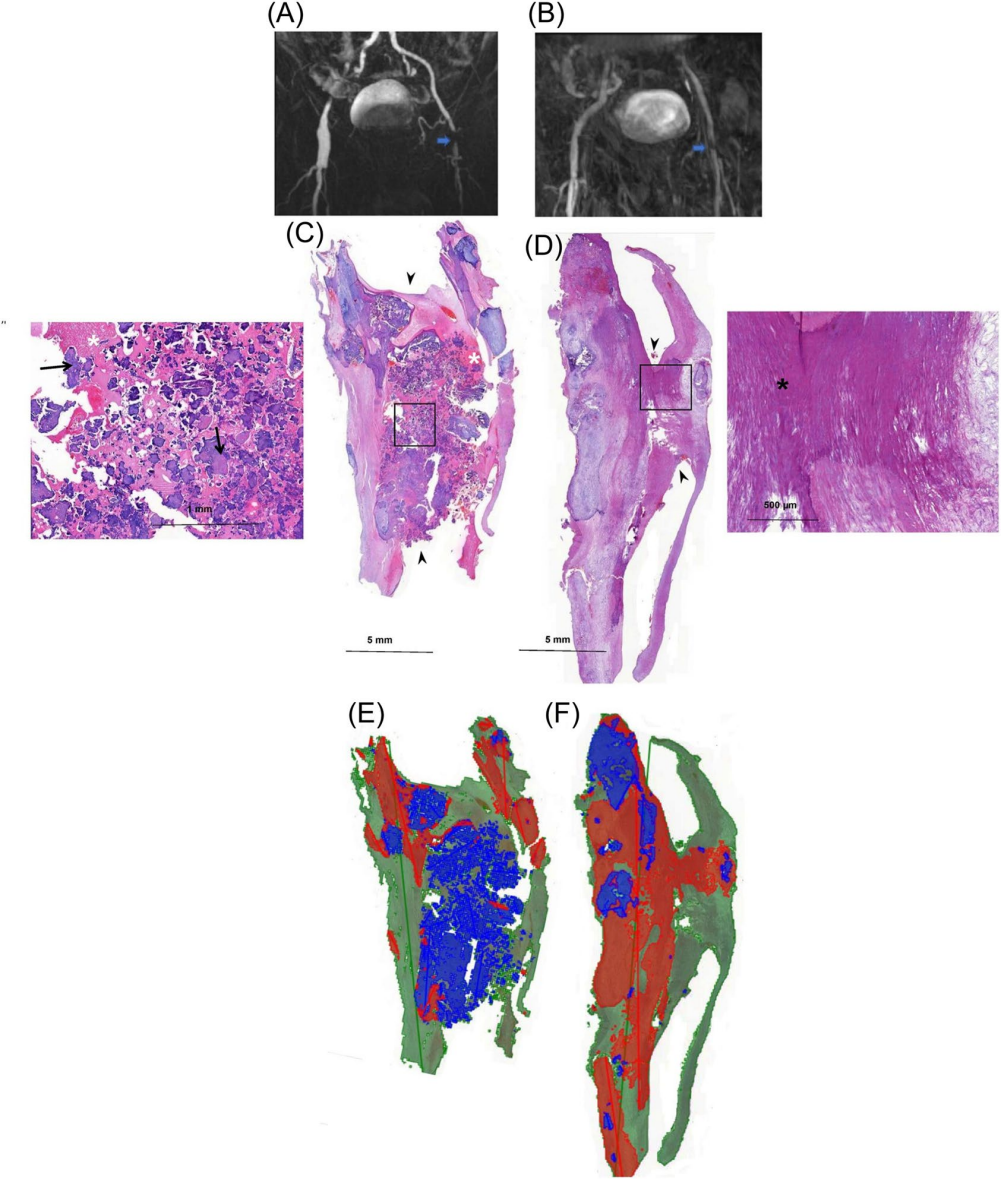
Obstructive femoral samples from diagnostic images to quantified histomorphometry. **A** and **B** are preoperative magnetic resonance images of two male patients that present obstructive level of stenosis of femoral artery (blue arrow) caused by the atherosclerotic lesions **C** and **D** respectively. Obstruction in these lesions is demonstrated histologically (arrowhead). Lesion **C** is dominated by nodular calcification, closely seen in the magnified image (thin arrow) surrounded by fibrin (white asterisk), while lesion **D** has mainly sheet calcification (black asterisk) in the magnified image. The trained algorithm (**E**) and (**F**) recognizes and calculates area proportion of nodular calcification (blue color) and sheet calcification (red color) to the sectioned plaque (green color) area. The calculated area proportion in **E** is 0.26 for nodular calcification and 0.10 for sheet calcification, while in **F** is 0.06 for nodular calcification and 0.40 for sheet calcification

Femoral plaque samples were collected during endarterectomy of the femoral artery bifurcation between October 2014 and January 2017 [4]. The severity of LEAD of the patients (*N* = 90) was determined by ankle brachial index (ABI), toe pressure (TP), Fontaine classification of LEAD symptoms, and urgency of the operation. Preoperative magnetic resonance angiography images were used to confirm the severity of the stenosis (Fig. 1).

We compared the area occupied by nodular calcification and sheet calcification in the plaque tissue to the clinical characteristics listed above. We analyzed endarterectomy samples, which fulfilled two criteria:1) more than 90% stenosis, including obstruction, was observed macroscopically, histologically and by magnetic resonance angiography, and 2) internal elastic lamina for vessel diameter measurement was identifiable in the plaques’ histological section indicating that the full diameter of the vessel was available for analysis.

Plaques were formalin-fixed, decalcified, and longitudinally sectioned into two halves. Sections were stained with Hematoxylin and eosin stain for histomorphometry analysis. Sample processing and laboratory analyses are described in detail elsewhere [4].

### Deep learning algorithm training

Hematoxylin and eosin-stained slides of femoral plaques longitudinal sections were digitized with a whole-slide scanner (3D HISTECH Pannoramic 250 Flash III, 3DHistec, Budapest, Hungary) with 20 × objective and a pixel size of 0.23 µm. The slides were then uploaded to a cloud-based image deep learning platform (Aiforia Create, Aiforia Technologies Oy, Helsinki, Finland, https://www.aiforia.com/).

To quantify each of the calcification categories, two sequential algorithms were developed; the first algorithm, the plaque tissue algorithm, recognized and quantified the area of plaque tissue from slide background. The algorithm was set to region context size of 50 µM, the complexity level of Complex and the default specifications. Upon this algorithm, a second algorithm, the calcification algorithm, was built for quantification of the calcification categories, nodular calcification and sheet calcification. While developing the algorithms, accuracy was assessed through 1) Verification of each annotation and 2) Analysis of the untrained regions and whole section slides. Calcification algorithm was fine adjusted on the following parameters: iterations = 7000, field of view = 100 x, image augmentation range (-10 to 10), aspect ratio = 1, maximum sheer = 1, luminance range (-30 to 30), contrast range (-30 to 30), maximum white balance change = 10, and noise = 2. The algorithm quantified nodular calcification and sheet calcification as the area of every recognized structure of the category in mm^2^, the collective area of each category, and the area proportion of the calcification category to that of the plaque section tissue area (Fig. 1).

The maximum width of the vessel diameter in the most stenosed part was measured to assess the vascular remodeling in relation to the area proportion of nodular calcification and sheet calcification. This was done using the measurement tool in the Aiforia platform (supplementary figure S4).

### Validation of the deep learning algorithm

To optimize the analysis, visual validation of the final algorithm analysis was conducted, whereafter, tiny lesions that were part of the continuum yet did not contribute to an actual nodule, were excluded. This was done after visually determining the cut-off size limit of the lesion per each slide.

In validation analysis, eight sections with highest amount of nodular calcification (> 35%) and eight sections with highest amount of sheet calcification (> 25%) were identified. Validation areas (approximately 500 µm × 1000 µm) containing both categories were selected in each section and selected for analysis by one investigator (IL). A validating investigator (MIM), unaware of the regions defined by the algorithm, defined independently regions of nodular calcification and sheet calcification within the validation areas. The data was analyzed by comparing the performance of the analysis algorithm to the allocation of calcification categories by the validator in the validation areas.

### Data analysis

Area proportions of nodular calcification and sheet calcification in plaque tissue were analyzed in relation to patients’ continuous and categorized binomial variables. Continuous data analysis of the patients is presented as the mean (± standard deviation). Data were analyzed for normal distribution by Shapiro–Wilk test. Normally distributed data were analyzed by two-tailed t-test and Pearson correlation test, while non-normally distributed data were analyzed by Mann–Whitney U and Spearman rank analysis. Analysis of covariance assessed association of measured vessel diameter with area proportion of nodular calcification and sheet calcification along with other confounding factors; age, gender, body mass index (BMI), smoking, hypertension, glomerular filtration rate (GFR) and inflammatory state (high sensitivity C-reactive protein (hs-CRP)). Our data fit the assumption of logistic regression analysis for the association of area proportion of nodular calcification and sheet calcification with the clinical parameters of LEAD, adjusted by hypertension, diabetes and dyslipidemia. P value < 0.05 was considered statistically significant. Data were analyzed using SPSS 25 (Armonk, NY: IBM Corp).

The age was categorized by the median into patients older or younger than 70.5 years. BMI was categorized by the cutoff point of normal and overweight (25 kg/m^2^) into normal, and overweight or obese (combined). Laboratory measurements were categorized according to the standardized age/gender-relevant reference values that are adopted in the analyzing facility, Helsinki University Hospital Laboratory Services (HUSLAB). Hs-CRP was considered increased for females when levels exceeded 2.5 mg/L and for males when levels exceeded 3 mg/L. GFR was considered impaired if it was less than the following values measured in ml/min /1.73 m^2^: 77 for patients aged 50–59 years, 69 for patients aged 60–69 and 59 for patients aged 70 years and older. Leukocytosis was labelled for readings higher than 8.2 E9/L, while anemia was deduced from females’ hemoglobin readings < 117 g/L and from males’ readings < 134 g/L. The categorization of ABI and TP values were based on clinical guidelines recommendations [14]. ABI less than 0.4 and TP less than 30 mmHg were deemed as severe disease indicators. Fontaine class was categorized into two groups, patients with claudication were deemed to have mild symptoms and patients with rest pain, ischemic ulcer or gangrene were considered to have severe symptoms [4]. Surgical interventions were classified into elective operations, semi-urgent operations were determined if surgical intervention was required within 4 weeks of the clinical evaluation.

## Results

The precision of the finalized algorithm was 97.53%, sensitivity was 97.66% and total area error (false positive and negative) was 2.06%. Validation analysis indicated that the algorithm was successful in identification of nodular calcification (error 0.23%) and sheet calcification (0.61%, supplementary Table S3, Supplementary figure S5).

Patients’ characteristics are shown in Table 1. Area proportion of sheet calcification correlated positively with serum HDL cholesterol (*R* = 0.419, *p* < 0.005), and negatively with serum LDL cholesterol (*R* = -0.346, *p* < 0.05), and serum triglyceride levels (*R* = -0.371, *p* < 0.01; supplementary Table S2). Overweight patients (BMI > 25) had higher area proportion of sheet calcification than lean patients (BMI < 25, 20.0 ± 15.1% vs 10.5 ± 6.9%, respectively, *p* < 0.05, Supplementary Table S1). Proportion of nodular calcification area to plaque tissue on the slide sections did not associate with any of the patient’s baseline characteristics.

**Table 1:** Patients’ characteristics

Characteristic	Total
N	51
Sections containing nodular calcification	49 (96)
Sections containing sheet calcification	48 (94.1)
Average nodular calcification area proportion (%)	22.4 ± 12.3
Average sheet calcification area proportion (%)	14.5 ± 11.8
Age (years) (*N* = 51)	70.5 ± 6.9
Risk factors	
Sex: male	28 (54.9)
Diabetes type I or II	14 (28.6)
Hypertension	43 (84.3)
Dyslipidemia	45 (88.2)
Increased hs-CRP^a^	13 (25.5)
BMI = 25–30 (kg/m^2^)	18 (35.3)
BMI > 30	11 (21.6)
Smoking status (*N* = 49)	
Never	2 (4.0)
Current smoker	28 (54.9)
Ex- smoker	19 (37.3)
Co-morbidities	
Coronary artery disease (*N* = 49)	12 (24.5)
Cerebrovascular disease	3 (6.1)
Medications	
ACE inhibitors / AT blockers	38 (74.5)
Aspirin	36 (70.6)
Clopidogrel	7 (13.7)
Statins	38 (74.5)
Warfarin	3 (5.9)
Lab parameters	
Hemoglobin (g/L) (*N* = 50)	139.0 ± 13.9
Total leukocyte count (10E9/L) (*N* = 50)	7.9 ± 1.8
Thrombocytes (10E9/L) (*N* = 50)	267.2 ± 65.6
Total cholesterol (mmol/L)	4.3 ± 1.3
LDL-cholesterol (mmol/L) (*N* = 50)	2.1 ± 0.8
HDL-cholesterol (mmol/L)	1.3 ± 0.5
Triglycerides (mmol/L)	1.8 ± 2.3
LEAD symptoms severity (Fontaine class)	
Claudication (II, IIA, IIB)	34 (66.6)
Rest pain (III)	12 (23.5)
Ischemic ulcer or gangrene (IV)	5 (9.8)
Classification of the intervention	
Semiurgent operation	9 (17.6)
Elective operation	42 (82.4)
TP readings (*N* = 43)	
TP < 30 mmHg	12 (72.1)
TP ≥ 30 mmHg	31 (27.9)
ABI (*N* = 41)	
ABI < 0.4	19 (45.2)
ABI ≥ 0.4	23 (54.8)

Data presented as mean ± SD or count (%), data obtained from the 51 patients, otherwise, N represents the number of data available

*Abbreviations*: *BMI* Body mass index, *hs-CRP* High-sensitivity C-reactive protein, *GFR* Glomerular filtration rate, *ACE* Angiotensin-converting enzyme, *AT* Angiotensin II, *LDL* Low-density-lipoprotein, *HDL* High-density-lipoprotein, *LEAD* Lower extremity arterial disease, *TP* Toe pressure, *ABI* Ankle brachial index

^a^Females’ values > 2.5 mg/L and males’ values > 3 mg/L

Analysis of covariance for the association of vessel diameter to the relative amount of nodular calcification and sheet calcification along with other confounding factors; age, gender, BMI, smoking, hypertension, diabetes, GFR, and hs-CRP showed that the area proportion of nodular calcification associated significantly to the vessel diameter (F = 5.700, *p* < 0.05), however, sheet calcification association was non-significant (F = 2.43, *p* = 0.626, Table 2).

**Table 2:** Analysis of covariance of Nodular calcification and Sheet calcification area proportions in association to the vessel diameter

Category	Nodular calcification	Sheet calcification
N	49	48
F	10.19	5.282
*p*	** < 0.005**	** < 0.05**
F^a^	6.226	4.776
*p*^a^	** < 0.05**	** < 0.05**
F^b^	5.700	2.43
*p*^b^	** < 0.05**	0.626

*Abbreviations*: *F* F-value of variancem, *Nodular calcification* Nodular calcification area proportion, *Sheet calcification* Sheet calcification area proportion

^a^adjusted by demographic determinants of vessel diameter: gender, age and BMI

^b^adjusted by modulants of vascular remodeling: smoking, hypertension, diabetes, renal impairment (GFR), and inflammatory state (hs-CRP), along with the demographic factors, gender, age and BMI

**Fig. 2:**
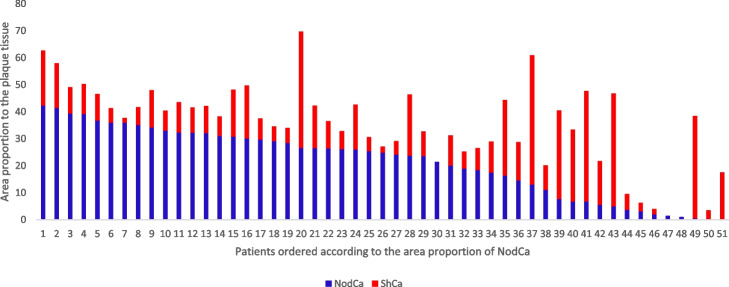
The collective area proportion of nodular calcification and sheet calcification of the 51 patients with > 90% stenosis included in the study

Proportion of nodular calcification and sheet calcification areas on each individual slide sections are shown in Fig. 2. Higher Nodular calcification area proportion associated with reduced likelihood of having severely lowered TP (< 30 mmHg), (OR = 0.903, 95% CI = 0.843–0.967, *p* < 0.005) and severely lowered ABI (< 0.4) (OR = 0.925, 95%CI = 0.873–0.980, *p* < 0.001). Multivariate logistic analysis including independently associating variants, i.e., age, gender, hypertension, diabetes and dyslipidemia along with nodular calcification showed that nodular calcification association remained significant; (OR = 0.910, 95% CI = 0.835–0.992, *p* < 0.05), (OR = 0.912, 95% CI = 0.84–0.986, *p* < 0.05) for TP and ABI, respectively. Additionally, higher nodular calcification area proportion was associated with reduced risk of semi-urgent operation (OR = 0.924, 95% CI = 0.868–0.984, *p* < 0.05), adjusted analysis to the above-mentioned confounders was also significant, (OR = 0.882, 95% CI = 0.797–0.976, *p* < 0.05). Higher nodular calcification area proportion was not associated with Fontaine class III, IV (rest pain and ischemic ulcer). Sheet calcification area proportion was not associated with any of the mentioned LEAD severity indicators (Table 3).

**Table 3:** Binary logistic regression of LEAD severity indicators in association with nodular calcification and sheet calcification area proportions

Category	Nodular calcification			Sheet calcification		
	OR	95% CI	*p*	OR	95% CI	*p*
Fontaine class III, IV	0.975	0.931–1.021	0.285	1.020	0.970–1.072	0.446
	^a^0.965	0.915–1.019	0.201	1.012	0.961–1.067	0.651
	^b^0.948	0.890–1.011	0.101	1.028	0.965–1.095	0.388
TP < 30 mmHg	0.903	0.843–0.967	** < 0.005**	1.008	0.949–1.071	0.797
	^a^0.913	0.846–0.985	** < 0.05**	1.015	0.948–1.086	0.673
	^b^0.910	0.835–0.992	** < 0.05**	0.997	0.92–1.075	0.938
ABI < 0.4	0.925	0.87–0.980	** < 0.01**	1.025	0.972–1.080	0.360
	^a^0.927	0.870–0.988	** < 0.05**	1.023	0.969–1.079	0.413
	^b^0.912	0.84–0.986	** < 0.05**	1.034	0.96–1.109	0.347
Semi-urgent operations	0.914	0.843–0.992	** < 0.05**	1.031	0.969–1.096	0.339
	^a^0.904	0.841–0.972	** < 0.01**	1.060	0.998–1.126	0.058
	^b^0.882	0.797–0.976	** < 0.05**	1.057	0.985–1.135	0.125

*Abbreviations*: *LEAD* Lower extremity arterial disease, *Nodular calcification* Nodular calcification area proportion, *Sheet calcification* Sheet calcification area proportion, *TP* toe pressure, *ABI* Ankle brachial index

^a^adjusted by gender and age

^b^adjusted by gender, age, BMI, diabetes, hypertension and dyslipidemia

## Discussion

We have, for the first time, quantified the amount of nodular calcification and sheet calcification on femoral artery plaque sections. The results revealed that higher amount of nodular calcification is associated with reduced ischemia of the lower limb, even at over 90% level of stenosis. Additionally, higher relative amount of nodular calcification is associated with probability of an elective operation. The latter implicates a milder LEAD presentation.

Nodular calcification and sheet calcification typically coexist in the same plaque. To understand their clinical relevance, the quantification of nodular calcification and sheet calcification lesions is needed. Quantifying of multiple features accurately and consistently is difficult using the conventional scoring method. To gain precision and objectivity, we applied a deep learning algorithm analysis for the localization and quantification of these calcified structures. The quantification was validated by the high sensitivity and specificity, with approximately 2% area error. In validation by an independent pathologist, < 1% area error was recorded in comparison to the calcification category allocation by the algorithm, further corroborating the analysis.

Nodular calcification has been suggested to emerge by fracture from sheet calcification [15]. This possibly explains the highest prevalence of nodular calcification in the superficial femoral artery, which is subjected to most dynamic movement, in comparison to more stagnant coronary and carotid arteries [10]. Furthermore, smooth muscle cells of femoral artery have an inherently different molecular makeup from other vascular beds; their higher expression of TGFβ promotes mineralizing activity [16]. This may explain the dense calcification recorded in this study.

All study samples were obtained from operated patients with total femoral artery occlusion or > 90% stenosis in the preoperative magnetic resonance angiography. High grade stenotic lesions were confirmed at specimen gross processing and histologically. Interestingly, distal limb perfusion in these patients, quantified by measurements of toe pressure and ankle brachial index, varied in their severity. This clinical variation is reflected in the amount and relative quantity of the calcified structures in the patients’ arteries. In our data, however, the observed clinical variation was mainly associated to the quantity of nodular calcification.

The longitudinally sectioned plaques are suitable for this analysis, since they give access to the whole length of the lesion structure, which allows for a more reliable quantification than analysis of horizontally sectioned plaques. Consequently, nodular calcification was encountered in 96% of sections with over 90% level of stenosis. Despite the high prevalence of nodular calcification in the studied obstructed/semi-obstructed lesions, quantification of nodular calcification in these samples has revealed that its relative amount contributed to a milder presentation of LEAD. Nodular calcification negatively associated with severely lowered ABI and TP. Furthermore, higher relative amount of nodular calcification was associated with decreased risk of semi-urgent intervention. No association was observed between the amount of sheet calcification and the clinical presentation of LEAD.

The precise quantifying approach has demonstrated that the increase of the relative amount of nodular calcification contributed significantly to the increase of the measured vessel diameter in samples with over 90% stenosis. Larger arteries at the obstructive/semi-obstructive level may incubate larger plaques and likely more nodular calcification. The quantification in this study is made relative to the plaque area. Furthermore, the analyzed association, after adjustment to the demographic determinants of vessel size: gender, age and BMI; and modifiers of vascular remodeling: smoking, hypertension, diabetes, GFR, and hs-CRP, remained significant [17, 18]. This indicates that nodular calcification may have some contribution to an expansive vascular remodeling. No association was noted between the amount of sheet calcification and the measured vessel diameter in these samples.

We postulate, that in comparison to rigid arteries with atherosclerosis characterized by sheet calcification, the prevalence of nodular calcification offers more flexibility of the artery structure. Flexibility allows for expansion of the artery and milder disease presentation. The loss of the compact calcified structure by the fragmentation of nodular calcification, and the separation of nodular calcification fragments by fibrin that collects from the leaky capillaries may enhance the expansive vascular remodeling [15]. Expansive vascular remodeling is proved to delay stenosis progression [19]. This possibly indicates a slower obstruction of vessels with abundant nodular calcification in their lesions [19]. This may reflect in our results as a less severe clinical presentation of lower limb ischemia.

To our knowledge, there are no previous studies on the association of quantified nodular calcification to clinical data in LEAD. However, the presence of calcified nodules on femoral plaque samples has been observed to protect against post-operative major amputation and/or re-intervention of the revascularized limb [20]. In the current study, the patients were not analyzed post-operatively.

The amount of sheet calcification was significantly reduced in patients with normal BMI in comparison to overweight/obese patients. Conforming with this finding, the amount of sheet calcification inversely correlated with serum levels of LDL cholesterol and triglyceride, and positively correlated with serum levels of HDL cholesterol. Increased concentration of lipids in serum is believed to mainly enhance the deposition of early calcification in the form of microcalcification, as corroborated by previous histological studies [21–23].

The present study has some limitations. The generalizability of the developed algorithm may be limited because the stainings were all done in one laboratory limiting color variation, all slides were scanned with one scanner, algorithm teaching and analysis was performed using a single 4-μm-thick longitudinal section from the thickest part of each plaque harvested from the bifurcation of the common femoral artery, thereby limiting the samples’ representability of the three-dimensional plaque structure. Furthermore, different arterial beds have reportedly different morphological features also in the atherosclerotic lesions [9]. However, we have shown the capability of the algorithm to analyze specimens with great accuracy, and the algorithm may be developed more generalizable by using new and more varied teaching slides. The small sample size limits the strength of statistical analysis. Clinical characteristics were retrospectively collected from the electronic patient records and included therefore only those details concerning each patient that had been documented.” The strength of the study is the wealth of rigorous clinical pre-operative data. We developed a reliable neural network method to quantify different forms of arterial calcification. The trained algorithm attained high sensitivity and specificity. The study material consisted of plaques harvested during surgical intervention; therefore, they represent a later stage of the disease. Our findings linking nodular calcification and milder disease, yet increased vessel diameter represent the first cross-sectional study to quantify the relative amount of nodular calcification and sheet calcification.

## Conclusion

Femoral plaques with over 90% stenosis demonstrate variable clinical severity of LEAD. The applied deep learning analyzing method allows for categorical quantification of calcification types in the plaque’s sections. The quantifying approach is more reliable than simply indicating the presence of calcification category in terms of clinical significance assessment. The quantifying approach for calcification categories has revealed that the relative predominance of nodular calcification indicates a slowly progressing obstruction diagnosed by alleviated ABI and TP readings and reduced semi-urgent surgical intervention and a possible expansive vascular remodeling. These results improve our understanding of the complex pathophysiology of LEAD and pave the way for personalized treatment options targeted at the specific pathological findings of patients with atherosclerotic disease.

## Supplementary Information

**Additional file 1.**

## Data Availability

The datasets used and/or analyzed during the current study are available from the corresponding author on reasonable request.

## Abbreviations

LEAD: Lower extremity arterial disease
BMI: Body mass index
Hs-CRP: High-sensitivity C-reactive protein
GFR: Glomerular filtration rate
LDL: Low-density-lipoprotein
HDL: High-density-lipoprotein
TP: Toe pressure
ABI: Ankle brachial index
ACE: Angiotensin-converting enzyme
AT: Angiotensin II

## References

[1] Chowdhury MM, Makris GC, Tarkin JM, Joshi FR, Hayes PD, Rudd JHF, et al. Lower limb arterial calcification (LLAC) scores in patients with symptomatic peripheral arterial disease are associated with increased cardiac mortality and morbidity. PLoS One. 2017;12(9):e0182952. 10.1371/journal.pone.0182952 PMC5590737 28886041

[2] Zettervall SL, Marshall AP, Fleser P, Guzman RJ (2018). Association of arterial calcification with chronic limb ischemia in patients with peripheral artery disease. J Vasc Surg.

[3] Pugliese G, Iacobini C, Fantauzzi CB, Menini S (2015). The dark and bright side of atherosclerotic calcification. Atherosclerosis.

[4] Laivuori M, Tolva J, Lokki AI, Linder N, Lundin J, Paakkanen R (2020). Osteoid Metaplasia in Femoral Artery Plaques Is Associated With the Clinical Severity of Lower Extremity Artery Disease in Men. Front CardiovascMed.

[5] Cardoso L, Weinbaum S (2018). Microcalcifications, their genesis, growth, and biomechanical stability in fibrous cap rupture. Adv Exp Med Biol.

[6] Puri R, Nicholls SJ, Shao M, Kataoka Y, Uno K, Kapadia SR (2015). Impact of statins on serial coronary calcification during atheroma progression and regression. J Am Coll Cardiol.

[7] Otsuka F, Yasuda S, Noguchi T, Ishibashi-Ueda H (2016). Pathology of coronary atherosclerosis and thrombosis. Cardiovasc Diagn Ther.

[8] Pappachan JM, Bino BC (2007). Calcified left atrial thrombus. N Engl J Med.

[9] Herisson F, Heymann MF, Chétiveaux M, Charrier C, Battaglia S, Pilet P (2011). Carotid and femoral atherosclerotic plaques show different morphology. Atherosclerosis.

[10] Torii S, Mustapha JA, Narula J, Mori H, Saab F, Jinnouchi H, et al. Histopathologic Characterization of Peripheral Arteries in Subjects With Abundant Risk Factors. JACC: Cardiovascular Imaging; 2018. 10.1016/j.jcmg.2018.08.039 30553660

[11] Ohtake T, Oka M, Ikee R, Mochida Y, Ishioka K, Moriya H (2011). Impact of lower limbs’ arterial calcification on the prevalence and severity of PAD in patients on hemodialysis. J Vasc Surg.

[12] Poredos P, Poredos P, Jezovnik MK (2018). Structure of atherosclerotic plaques in different vascular territories: clinical relevance. Curr Vasc Pharmacol.

[13] Birnbaum Y, Fishbein MC, Luo H, Nishioka T, Siegel RJ (1997). Regional remodeling of atherosclerotic arteries: a major determinant of clinical manifestations of disease. J Am Coll Cardiol.

[14] Conte MS, Bradbury AW, Kolh P, White JV, Dick F, Fitridge R (2019). Global vascular guidelines on the management of chronic limb-threatening ischemia. J Vasc Surg.

[15] Sakamoto A, Virmani R, Finn AV, Gupta A (2018). Calcified nodule as the cause of acute coronary syndrome: connecting bench observations to the bedside. Cardiology (Switzerland).

[16] Espitia O, Chatelais M, Steenman M, Charrier C, Maurel B, Georges S (2018). Implication of molecular vascular smooth muscle cell heterogeneity among arterial beds in arterial calcification. PLoS One.

[17] Hopkins ND, Green DJ, Tinken TM, Sutton L, McWhannell N, Thijssen DHJ (2009). Does conduit artery diameter vary according to the anthropometric characteristics of children or men?. Am J Physiol Heart Circ Physiol.

[18] Yang F, Minutello RM, Bhagan S, Sharma A, Wong SC (2006). The impact of gender on vessel size in patients with angiographically normal coronary arteries. J Interv Cardiol.

[19] Ward MR, Pasterkamp G, Yeung AC, Borst C (2000). Arterial remodeling. Circulation.

[20] Danvin A, Quillard T, Espitia O, Charrier C, Guyomarch B, Gouëffic Y (2019). Impact of femoral ossification on local and systemic cardiovascular patients’ condition. Ann Vasc Surg.

[21] Qunibi WY (2005). Dyslipidemia and progression of cardiovascular calcification (CVC) in patients with end-stage renal disease (ESRD). Kidney Int Suppl.

[22] Maldonado N, Kelly-Arnold A, Laudier D, Weinbaum S, Cardoso L (2015). Imaging and analysis of microcalcifications and lipid/necrotic core calcification in fibrous cap atheroma. Int J Cardiovasc Imaging.

[23] O’Brien KD, Reichenbach DD, Marcovina SM, Kuusisto J, Alpers CE, Otto CM (1996). Apolipoproteins B, (a), and E accumulate in the morphologically early lesion of “degenerative” valvular aortic stenosis. Arterioscler Thromb Vasc Biol.

