# Supplementary material for: High relative amount of nodular calcification in femoral plaques is associated with milder lower extremity arterial disease
Source: BMC Cardiovasc Disord. 2022 Dec 23;22:563. doi: 10.1186/s12872-022-02945-7 (PMC9783794; doi:10.1186/s12872-022-02945-7)
Supplement: Supplementary file 1 — Additional file 1. [file 12872_2022_2945_MOESM1_ESM.docx]

**Supplementary Table S1**

Association of NodCa and ShCa area proportions with the categorical baseline characteristics.

| Category | (n) NodCa | (n) ShCa | NodCa | ShCa | Category | (n)  NodCa | (n) ShCa | NodCa | ShCa | *p* NodCa | *p*  ShCa |
| --- | --- | --- | --- | --- | --- | --- | --- | --- | --- | --- | --- |
| Age ≥ 70.5 years | 22 | 22 | 22.6 ± 12.3 | 14.2 ± 11.9 | Age < 70.5 | 27 | 26 | 22.2 ± 12.5 | 14.8. ± 12.0 | 0.747 | 0.852 |
| Males | 28 | 26 | 23.9 ± 11.8 | 13.1 ± 10.0 | Females | 21 | 22 | 20.6 ± 12.9 | 16.2 ± 13.7 | 0.315 | 0.983 |
| BMI > 25 Kg/m^2^ | 28 | 26 | 24.7 ± 9.8 | 10.5 ± 6.9 | BMI (18.5-25) | 19 | 20 | 19.3 ± 15.4 | 20.0 ± 15.1 | 0.190 | **< 0.05** |
| Previous, current smokers | 45 | 44 | 22.3 ± 12.1 | 13.6 ± 11.3 | Never smokers | 2 | 2 | 17.4 ± 17.5 | 24.8 ± 24.1 | 0.578 | 0.495 |
| Diabetes type I or II | 14 | 13 | 26.4 ± 8.8 | 10.4 ± 5.5 | No | 33 | 33 | 20.6 ± 13.4 | 16.3 ± 13.5 | 0.085 | 0.400 |
| Hypertension | 41 | 41 | 22.4 ± 12.0 | 14.7 ± 11.6 | No | 8 | 7 | 22.3 ± 14.5 | 13.2 ± 13.7 | 0.971 | 0.492 |
| Dyslipidemia | 45 | 43 | 22.6 ± 12.1 | 13.7 ± 10.7 | No | 5 | 4 | 18.7 ± 14.5 | 25.1 ± 20.1 | 0.503 | 0.307 |
| Coronary artery disease | 12 | 12 | 26.2 ± 11.4 | 14.6 ± 12.0 | No | 35 | 34 | 20.8 ± 12.9 | 14.1 ± 12.1 | 0.305 | 0.755 |
| Cerebro-vascular accident | 3 | 3 | 28.6 ± 11.6 | 19.1 ± 9.2 | No | 44 | 43 | 22.5 ± 12.2 | 13.7 ± 11.7 | 0.406 | 0.208 |
| Increased hs-CRP^a^ | 13 | 12 | 26.9 ± 10.2 | 13.4 ± 11.5 | No | 36 | 36 | 20.8 ± 12.7 | 14.9 ± 12.0 | 0.121 | 0.775 |
| Impaired GFR^b^ | 9 | 9 | 28.5 ± 10.8 | 12.7 ± 11.8 | No | 39 | 38 | 21.6 ± 12.0 | 14.3 ± 11.5 | 0.119 | 0.604 |
| Leukocytosis^c^ | 19 | 19 | 24.0 ± 11.5 | 13.6 ± 11.4 | No | 27 | 26 | 10.5 ± 13.1 | 16.0 ± 12.6 | 0.357 | 0.513 |
| Anaemic^d^ | 7 | 12 | 23.8 ± 13.0 | 14.6 ± 12.0 | No | 41 | 34 | 22.0 ± 12.3 | 14.1 ± 12.1 | 0.728 | 0.608 |
| Statin users | 37 | 35 | 23.6 ± 11.7 | 14.9 ± 10.8 | No | 12 | 13 | 18.6 ± 13.6 | 13.6 ± 14.8 | 0.224 | 0.232 |

Association of NodCa and ShCa area proportions with the categorical baseline characteristics.

Data presented as mean ± SD; analysis of NodCa data by independent T-test; analysis of ShCa data by Mann-Whitney U test

Abbreviations: NodCa, nodular calcification; ShCa, sheet calcification; LEAD, lower extremity arterial disease; BMI, body mass index; hs-CRP, high sensitive chain reactive protein; GFR, glomerular filtration rate. No, absence of the category.

a Females’ values > 2.5 mg/L and males’ values > 3 mg/L.

b Values < 77 for patients aged (50-59)years, < 69 for patients aged (60-69), < 59 for patients aged 70 years and over.

c Values > 8.2 E9/L.

d Females’ Hb readings <117 g/L and males’ readings < 134 g/L.

**SupplementaryTable S2**

Correlation of NodCa and ShCa area proportions with the continuous patients’ characteristics.

|  | NodCa |  |  | ShCa |  |  |
| --- | --- | --- | --- | --- | --- | --- |
|  | rho | N | *p* | rho | N | *p* |
| Age (years) | 0.130 | 49 | 0.373 | 0.066 | 49 | 0.655 |
| BMI (kg/m^2^) | 0.094 | 47 | 0.531 | -0.181 | 46 | 0.23 |
| Leukocytes (10E9/L) | 0.002 | 48 | 0.989 | 0.042 | 47 | 0.777 |
| Hemoglobin (g/L) | -0.195 | 48 | 0.185 | 0.234 | 47 | 0.114 |
| Thrombocytes count (10E9/L) | -0.163 | 48 | 0.267 | -0.216 | 47 | 0.144 |
| hs-CRP (mg/L) | 0.200 | 49 | 0.168 | -0.099 | 48 | 0.505 |
| GFR (mL/min/1.73 m^2^) | -0.149 | 48 | 0.314 | 0.083 | 47 | 0.58 |
| Total-cholestrol (mmol/L) | 0.013 | 49 | 0.930 | -0.065 | 48 | 0.658 |
| LDL-cholesterol (mmol/L) | -0.017 | 48 | 0.907 | -0.346^*^ | 47 | **< 0.05** |
| HDL-cholestrol (mmol/L) | -0.078 | 49 | 0.594 | 0.419^**^ | 48 | **< 0.005** |
| Triglyceride (mmol/L) | 0.036 | 49 | 0.804 | -0.371^**^ | 48 | **< 0.01** |

NodCa data analysed by Pearson, ShCa data analysed by Spearman rank, rho is the correlation coefficient.

Abbreviations: NodCa, nodular calcification; ShCa, sheet calcification; BMI, body mass index; hs-CRP, high sensitive chain reactive protein; GFR, glomerular filtration rate.

**Supplementary Table S3**

(A) Comparison of the performance of the algorithm in annotated versus not annotated slides indicates that the mean area of nodular and sheet calcification on the sections is similar regardless of whether the samples were included in the algorithm training. The annotation process typically covers regions of interest of varying size and number in a particular section, never the full slide.

(B) Results of the independent validation was compared to the algorithm and the statistical results of the comparison are shown. Based on visual comparison (Supplementary Figure S5) and the statistics provided the validation was deemed successful thereby corroborating the precision of the analysis. It is notable that the area error % alone is not a suitable parameter for evaluating the performance of an image analysis algorithm without sufficient context. The validation tool used in this study compares which pixels in the whole slide image are seen calcified according to algorithm, as opposed to the markings made by human expert. Total area error % (sum of positive and negative area error%) in validation may increase if the algorithm marks the calcified objects with an area that overlaps with background (false positive area %) or does not fully mark them (false negative area %), or the human expert does unprecise validation markings. However, minute undulations at the borders of the marked calcifications do not change the fact that the calcifications were indeed detected and marked in consistent and highly reproducible manner by the algorithm.

(A)

|  |  | Mean Area (mm²) | Mean Area (%) |
| --- | --- | --- | --- |
| Nodular  calcification | annotated (N=29) | 37.89 | 22.95 |
|  | not annotated (N=18) | 42.39 | 23.45 |
| Sheet  calcification | annotated (N=29) | 24.70 | 15.08 |
|  | not annotated (N=18) | 17.39 | 12.07 |

(B)

|  | False positive (%) | False negative (%) | Error (%) | Precision (%) | Sensitivity (%) | F1 Score (%) |
| --- | --- | --- | --- | --- | --- | --- |
| Sheet calcification | 0,48 | 0,14 | 0,61 | 87,94 | 96,36 | 91,52 |
| Nodular calcification | 0,13 | 0,1 | 0,23 | 90,6 | 89,37 | 89,71 |


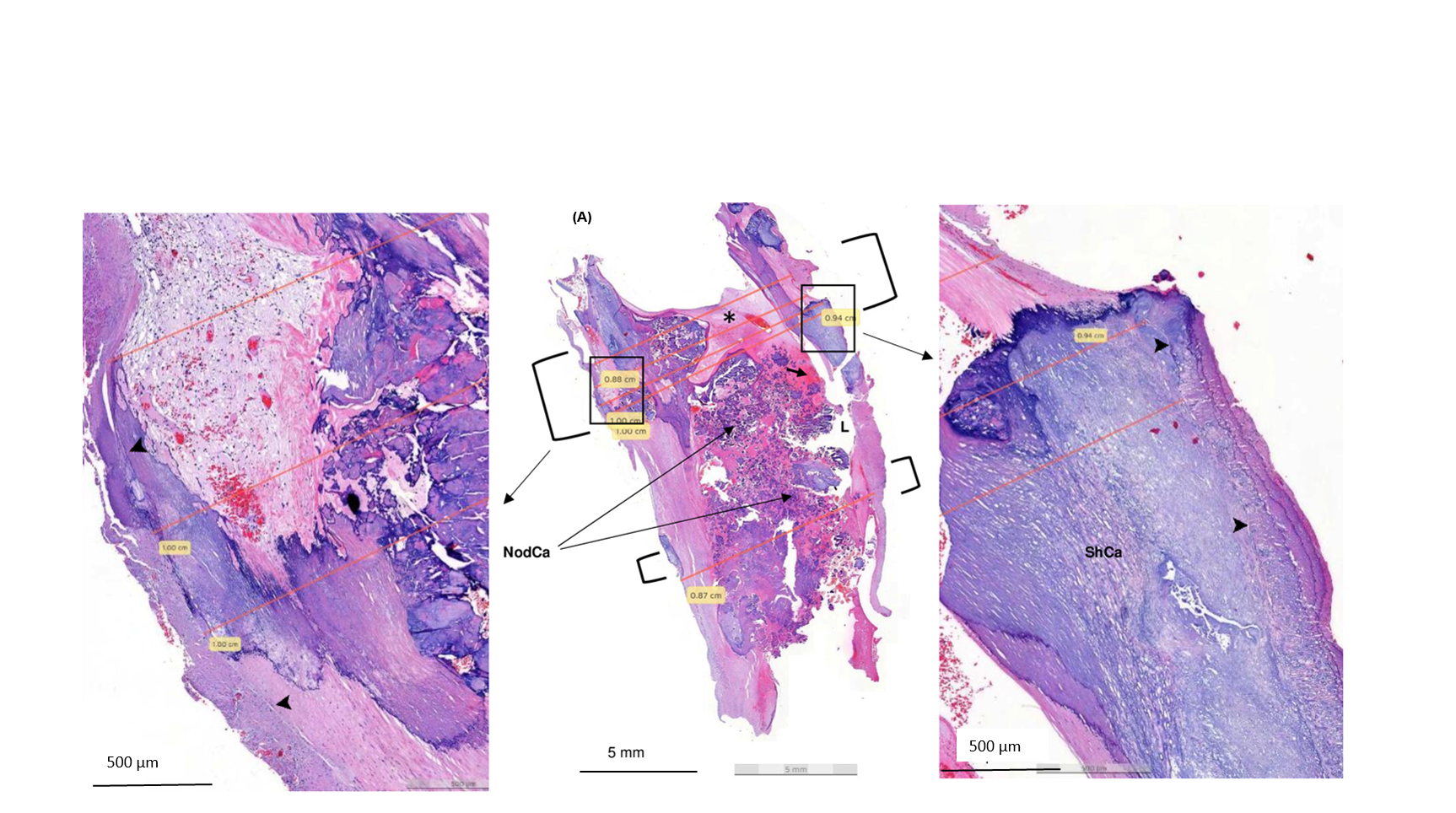


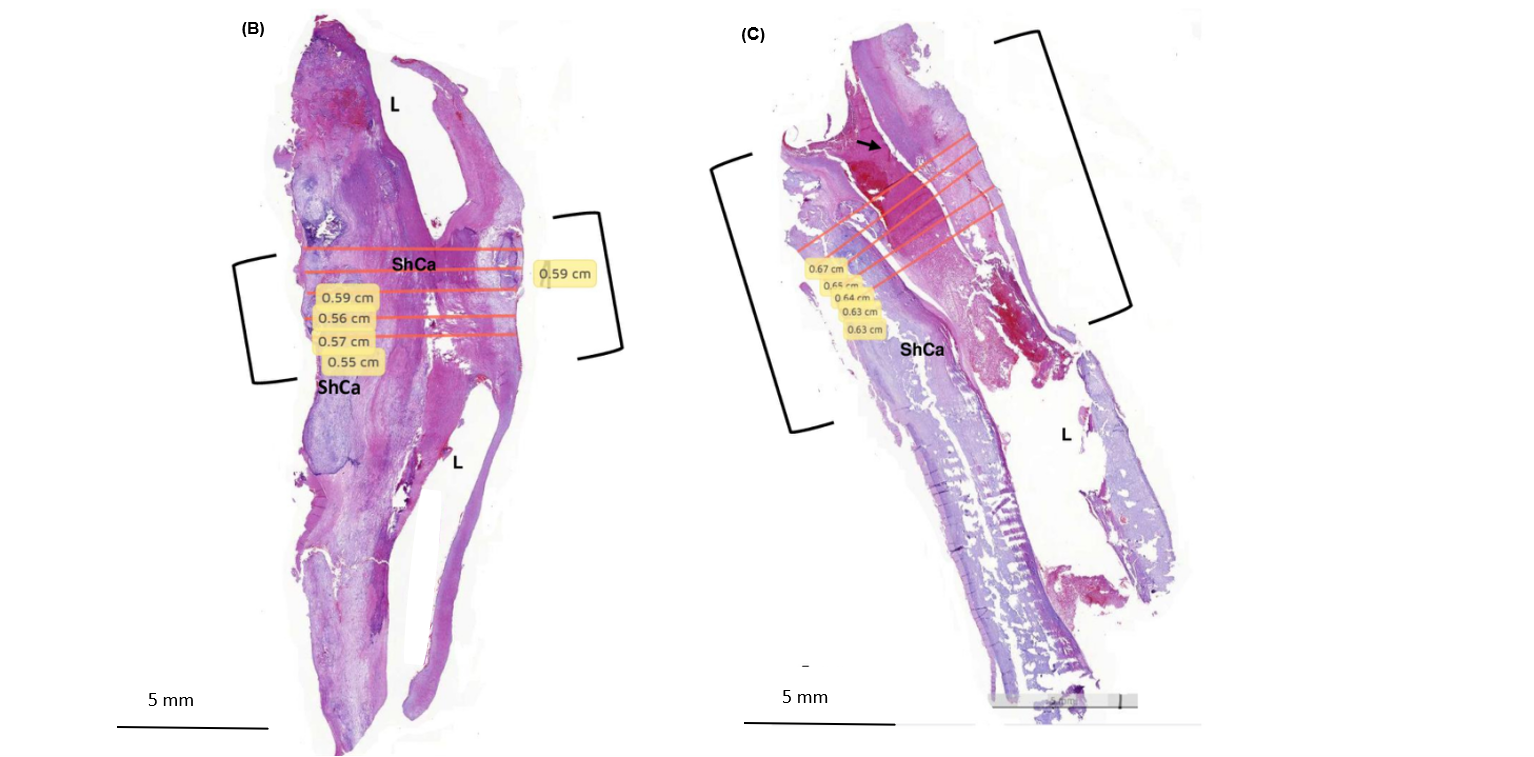


Supplementary Figure S4. Three examples of obstructed femoral plaques (A), (B) and (C) for illustration of the vessel diameter measurement. Maximum internal elastic vessel diameter at the obstruction site (surrounded by squared brackets) was measured. L indicates vessel lumen. Arrowhead refers to internal elastic lamina in the magnified subsections of plaque section (A). Section (A), has mainly nodular calcification (NodCa), 26% of the plaque tissue section, whereas (B) and (C) are dominated by sheet calcification (ShCa, 40% and 41% respectively. Section (A) has a larger measured vessel diameter(10mm) compared to section (B), 5.9mm and section (C), 6.7mm. Small arrow on (C) refers to fibrin, asterisk on (A) refers to the organized thrombus.

Supplementary Figure S4

Three examples of obstructed femoral plaques (A), (B) and (C) for illustration of the vessel diameter measurement. Maximum internal elastic vessel diameter at the obstruction site (surrounded by squared brackets) was measured. L indicates vessel lumen. Arrowhead refers to internal elastic lamina in the magnified subsections of plaque section (A). Section (A) has mainly nodular calcification, (Nodular calcification), 26% of the plaque tissue section, whereas (B) and (C) are dominated by sheet calcification (Sheet calcification, 40% and 41% respectively. Section (A) has a larger measured vessel diameter(10mm) compared to section (B), 5.9mm and section (C), 6.7mm. Small arrow on (C) refers to fibrin, asterisk on (A) refers to the organized thrombus.


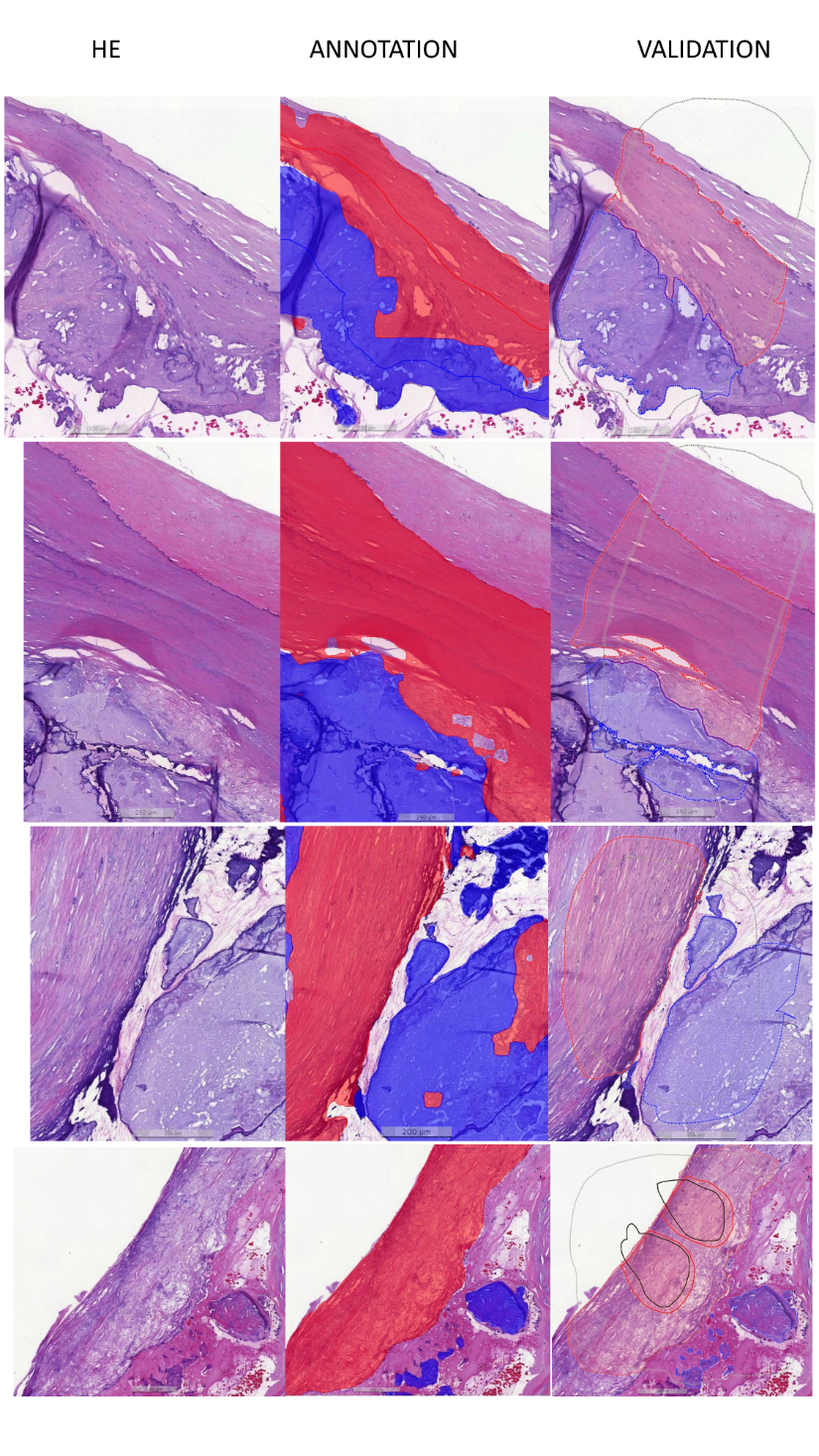
 Sample

1

2

3

4


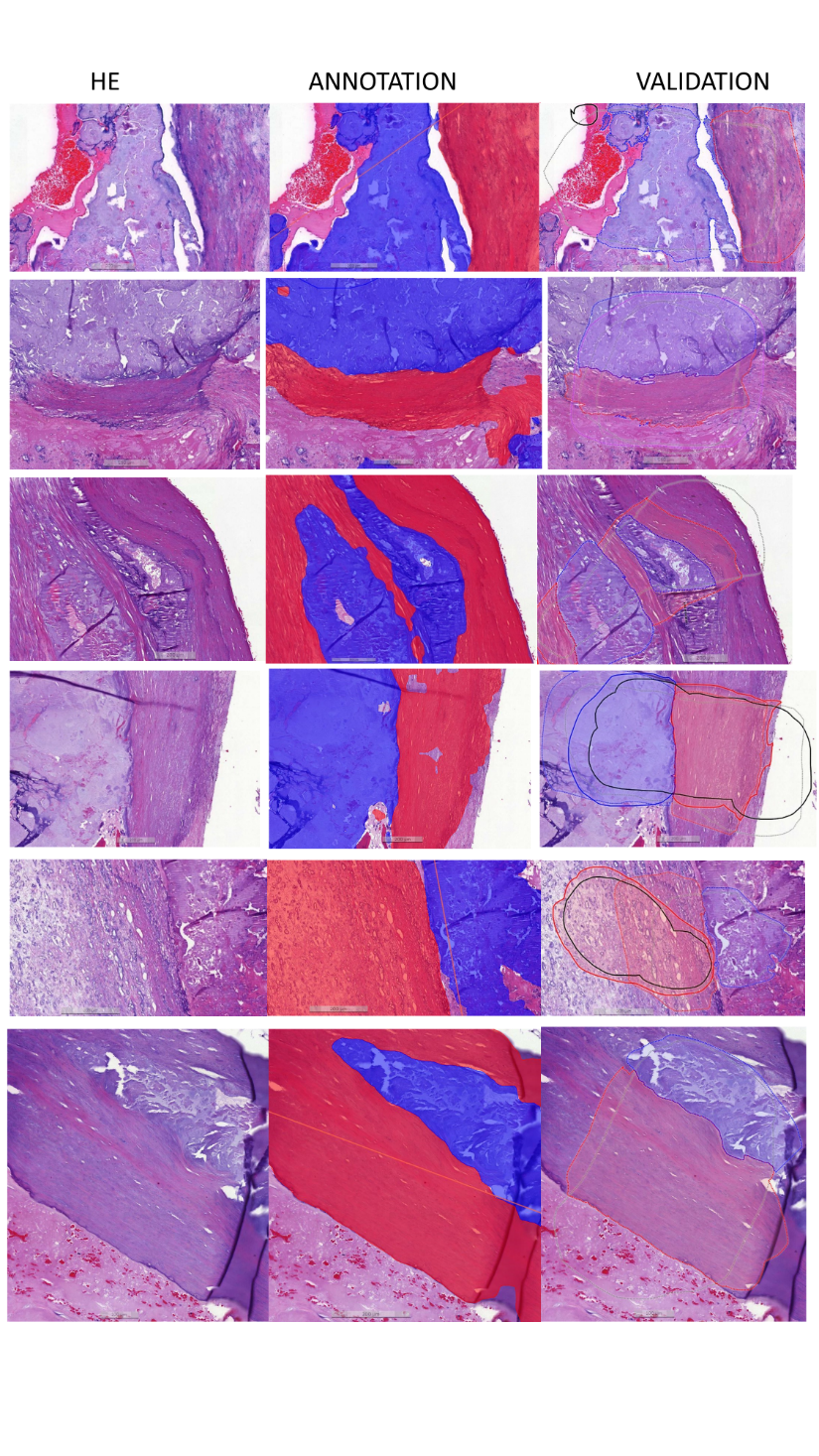


5

6

7

8

9

10

Supplementary Figure S5. Ten examples of annotation validation by an independent pathologist. Blue indicates sheet calcification and red nodular calcification. For each section, the left panel shows the HE stain of the analyzed region, in the middle panel is the same region after annotation by the finished algorithm and on the right panel is the validation analysis (validation region selected by light grey circle, validated sheet and nodular calcification by light blue and red area, respectively) by an independent pathologist. Black regions in right panel of 4, 5, 8, and 9 show examples of original algorithm annotation. In 5 the annotation represents a negative annotation for calcification. In cases 4, 8 and 9 the black annotation windows with blue/red shade show actual calcification annotations.
